# Gender and age differences in weekend eating habits: associations with fat mass percentage in a cross-sectional study

**DOI:** 10.3389/fnut.2025.1578574

**Published:** 2025-05-08

**Authors:** Stefania Gorini, Elisabetta Camajani, Alessandra Feraco, Andrea Armani, Chiara Quattrini, Maria Grazia Tarsitano, Edda Cava, Laura Di Renzo, Massimiliano Caprio, Antonino De Lorenzo, Mauro Lombardo

**Affiliations:** ^1^Department for the Promotion of Human Science and Quality of Life, San Raffaele Open University, Rome, Italy; ^2^Laboratory of Cardiovascular Endocrinology, San Raffaele Research Institute, IRCCS San Raffaele Roma, Rome, Italy; ^3^Clinical Nutrition and Dietetics, San Camillo Forlanini Hospital, Rome, Italy; ^4^Section of Clinical Nutrition and Nutrigenomic, Department of Biomedicine and Prevention, University of Rome Tor Vergata, Rome, Italy

**Keywords:** weekend eating habits, gender differences, age groups, fat mass percentage, body composition, dietary patterns, cross-sectional study

## Abstract

**Introduction:**

Weekend eating habits are often different from those of weekdays, but their impact on body composition remains little explored. This study investigates gender and age differences in weekend eating behaviors and their association with fat mass percentage (FM%).

**Methods:**

A cross-sectional study was conducted on 2,596 participants at an obesity center in Italy. Eating habits were assessed with a self-reported questionnaire, classifying weekend eating behavior into four categories: maintaining weekday eating patterns, cooking at home, eating out, and eating prepared meals. Weekday eating was considered the participant’s habitual eating pattern as reported during clinical evaluation. Differences in FM% between groups were analyzed by ANOVA and the influence of gender and age was examined by multiple linear regression models.

**Results:**

Significant differences between gender and age were observed specifically in weekend eating behaviors (*p* < 0.001). Women were significantly more likely to cook at home, whereas men, particularly those aged 18–30 years, were more likely to eat out. Women who cooked at home during the weekend had a higher FM% than those who ate out or maintained weekday eating habits (*p* < 0.001) but consistency alone does not guarantee better body composition, as the quality of the diet was not assessed. Among men aged 31–45 years, cooking at home was associated with a higher FM% than maintaining weekday habits (*p* = 0.0028). Regression analysis showed that FM% was higher in females and older age groups, while eating out, being hosted, or maintaining weekday habits were associated with lower FM% compared to cooking at home (all *p* < 0.05).

**Conclusion:**

Weekend eating habits represent a distinct and influential factor on body composition, rather than a simple extension of weekday patterns. The results emphasize that gender- and age-specific approaches are crucial in dietary interventions, particularly for younger men and women who maintain structured meal patterns. These results suggest that weekends may be an important period for dietary interventions based on self-reported dietary patterns, with potential implications for gender- and age-specific dietary interventions and broader public health strategies aimed at improving long-term metabolic outcomes.

## 1 Introduction

Eating behaviors, including meal times, food choices and eating environments, vary significantly by gender and age (1). While weekday meals tend to follow structured patterns dictated by work schedules and daily responsibilities, weekend eating habits are more flexible, influenced by cultural, social and psychological factors (2, 3). Previous research has shown that people tend to consume more calories, dine out more frequently and plan meals in a less structured manner at weekends than on weekdays (4, 5). However, the extent to which these behaviors differ between men and women in different age groups has not been sufficiently explored.

Gender differences in food choices are well-documented. Women generally report greater adherence to health-conscious diets, higher consumption of fruit and vegetables and more home-cooked meals (6). In contrast, men often consume more processed and high-calorie foods, have higher rates of skipping meals and rely more on external food sources such as restaurants or take-away (7). Age further modifies these behaviors: younger individuals favor convenience and socializing, while older adults often adhere to more stable and traditional eating patterns (8). However, specific weekend eating behaviors have received less attention in the literature, despite their potential impact on long-term nutritional status and metabolic health (9). Weekend eating habits are particularly relevant given their role in energy balance and overall diet quality. Studies have suggested that deviations from weekday eating patterns - often characterized by higher intakes of energy-dense foods and alcohol - may contribute to weight gain and metabolic dysregulation (10, 11). For instance, Zeballos and Todd (7) reported that adults in the United States consume on average 344 more kilocalories on weekends compared to weekdays, highlighting the potential impact of weekend dietary deviations. In addition, social consumption contexts, more common on weekends, may influence portion sizes and food choices, sometimes counteracting the benefits of structured weekday diets (3, 12–14). Understanding how gender and age interact to shape these behaviors is crucial for designing targeted nutritional interventions that take biological and sociocultural influences into account.

This study aims to examine gender- and age-related variations in weekend eating habits within a large sample, focusing on differences in meal preparation, meals out and dietary consistency between age groups. These four categories were selected on the basis of previous research indicating that weekend eating behavior often differs from weekday patterns and can be broadly classified into maintaining weekday habits, cooking at home, eating out and eating prepared meals. This classification makes it possible to examine the relationship between specific weekend food choices and body composition and metabolic health. By identifying distinct behavioral patterns, this research sheds light on how specific weekend food choices influence body composition and may contribute to metabolic disparities and long-term health risks.

## 2 Materials and methods

### 2.1 Participants

This cross-sectional study was conducted at an obesity center in Rome, Italy, where 3,000 medical records were initially screened to identify eligible participants. The final sample included 2,596 people, recruited between January 2023 and November 2024. Participants had to be adults over the age of 18, able to complete an online survey in Italian prior to the initial visit and willing to provide written informed consent. A power analysis was conducted to determine the minimum sample size required to detect significant differences in fat mass percentage (FM%) between weekend eating habits, adjusted for age and gender. Based on an expected effect of η^2^ = 0.02, a statistical power of 0.80 and an alpha level of 0.05, a sample size of approximately 2,000 participants was deemed necessary. Exclusions were applied to maintain data quality and reduce bias. Records were excluded if participants had missing or incomplete responses, implausible anthropometric values or diagnosed metabolic disorders that could influence eating behavior or body composition, such as type 2 diabetes, hypothyroidism, Cushing’s syndrome, or polycystic ovary syndrome (PCOS). Additional exclusions were made for subjects who reported unusual eating habits unrelated to the purpose of the study, such as strict medical diets or meal replacement regimes, as well as for those who did not consent to body composition assessment. All participants were recruited within the clinical setting of the obesity center during their initial evaluation. No external recruitment was conducted.

### 2.2 Questionnaire

The questionnaire, originally designed to capture a comprehensive profile of eating habits, was administered electronically prior to the participants’ initial visits. It was accessible from any device with an Internet connection, thus ensuring ease of completion. Although the complete questionnaire included several sections covering various aspects of eating behavior, taste preferences and physical activity, we only analyzed the answers to the question: “Do you eat differently at the weekend?”. Participants answered a single-choice question with four predefined options regarding their typical weekend eating behavior: (1) eating more elaborately, (2) eating out, (3) cooking elaborately at home, or (4) maintaining the same eating habits as on weekdays. This classification was based on patterns frequently observed in clinical practice and allowed for standardized grouping in the analysis. Although the full questionnaire has been used in previous studies (2), its structure remained consistent with validated eating behavior assessments (15). Responses were collected anonymously and participants provided consent before starting the survey.

### 2.3 Body composition

Participants underwent a standardized medical evaluation that included dietary history, physical examination and body composition assessment. Measurements were taken in the morning after an overnight fast, with participants wearing minimal clothing to ensure accuracy. Body weight was recorded with a calibrated electronic scale (Tanita BC-420 MA) placed on a solid, even surface. To minimize measurement error, each participant was weighed twice and, if discrepancies exceeded 100 g, a third reading was taken; the two closest values were used for analysis. Height was measured with a stadiometer, with the participants standing barefoot in an upright position, their heads aligned with the Frankfurt plane. Two measurements were recorded and, if the differences were greater than 0.1 cm, a third measurement was taken to ensure consistency. Body composition analysis was conducted by bioelectrical impedance analysis (BIA) using the Tanita BC-420 MA, a device validated against air displacement plethysmography (BodPod) for its accuracy (16). This method provided measurements of fat mass percentage, fat-free mass and total body water, with a sensitivity of 100 g. To standardize the conditions, participants were instructed to fast for at least 3 h before the assessment, to abstain from strenuous physical activity for 12 h and to avoid excessive consumption of food, liquids and alcohol the night before. Women were advised to schedule evaluations outside the menstrual period to minimize changes related to fluid retention. All evaluations were conducted by qualified professionals following a strict protocol to ensure reliability and comparability of data between subjects.

### 2.4 Statistical analysis

The data were analyzed using SPSS v. 28 (IBM Corporation, Armonk, NY, United States). Continuous variables were expressed as mean ± standard deviation (SD) and categorical variables as *n* (%). Comparisons between groups were performed using *t*-tests for normally distributed continuous variables, Mann-Whitney U-tests for non-normally distributed variables and chi-square (χ^2^) tests for categorical variables. To assess the effect of weekend eating habits on FM%, an ANOVA was conducted, followed by Tukey HSD *post-hoc* tests to determine significant differences between groups. A multiple linear regression model, adjusted for age, gender, BMI and income, was applied to assess associations between weekend eating habits and body composition parameters. Differences in demographic characteristics (age, gender, income, smoking status) between groups were tested using chi-square tests for categorical variables and *t*-tests or Mann-Whitney U-tests for continuous variables, as appropriate. Statistical significance was set at *p* < 0.05.

## 3 Results

The study included 2,596 participants (Table 1; 40.4% male, 59.1% female). The age distribution was significantly different (*p* = 0.0019), with the majority of participants aged between 31 and 45 years (40.2%). Males had a higher BMI (28.5 ± 5.5 vs. 27.2 ± 5.3 kg/m^2^, *p* < 0.001) and FM in kilograms (62.6 ± 8.3 vs. 44.2 ± 5.2 kg, *p* < 0.001), while females had a higher FM percentage relative to total body weight (FM%) (34.1 ± 8.0 vs. 24.4 ± 9.1%, *p* < 0.001). Smoking habits were similar between genders (*p* = 0.3460); however, these differences reflected minor variations across income brackets, without a consistent pattern indicating higher income for one gender over the other.

**TABLE 1:** Demographic and clinical characteristics of the study population (*n* = 2,596).

Variable	Unit	Total	Male	Female	*p*-value
Total sample	*n* (%)	2,596	1,062 (40.4%)	1,534 (59.1%)	0.0019
Age 18–30 years	*n* (%)	659 (25.3%)	296 (27.9%)	363 (23.7%)	
Age 31–45 years	*n* (%)	1,044 (40.2%)	441 (41.5%)	603 (39.3%)	
Age 46–60 years	*n* (%)	666 (25.8%)	250 (23.5%)	416 (27.1%)	
Age 60+ years	*n* (%)	227 (8.7%)	75 (7.1%)	152 (9.9%)	
Smokers	*n* (%)	619 (23.8%)	244 (23.0%)	375 (24.7%)	0.3460
BMI	kg/m^2^	27.7 ± 5.4	28.5 ± 5.5	27.2 ± 5.3	< 0.001
Fat mass	kg	24.3 ± 11.1	22.6 ± 11.6	25.5 ± 10.6	< 0.001
Fat mass	%	30.1 ± 9.7	24.4 ± 9.1	34.1 ± 8.0	< 0.001
Abdominal circumference	cm	95.9 ± 14.3	100.3 ± 14.6	92.9 ± 13.2	< 0.001
Fat-free mass	kg	51.7 ± 11.2	62.6 ± 8.3	44.2 ± 5.2	< 0.001
Yearly income < €20,000	*n* (%)	421 (16.2%)	144 (13.6%)	277 (18.1%)	0.0183
Yearly income €20,000–€40,000	*n* (%)	1,762 (67.9%)	740 (69.7%)	1,022 (66.8%)	
Yearly income €40,000–€60,000	*n* (%)	342 (13.2%)	150 (14.1%)	192 (12.6%)	
Yearly income > €60,000	*n* (%)	71 (2.7%)	28 (2.6%)	43 (2.8%)	

Data are presented as mean ± standard deviation (SD) for continuous variables and *n* (%) for categorical variables. Statistically significant differences between males and females were determined using the *t*-test for continuous variables and the chi-square test for categorical variables. The *p*-value indicates significant differences between the genders, where applicable.

**FIGURE 1:**
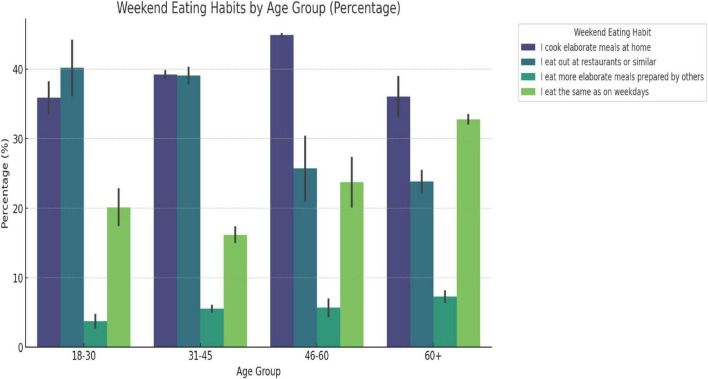
Weekend eating habits across age groups. This bar chart displays the overall distribution of weekend eating habits across all age groups, irrespective of gender. A chi-squared test confirms significant differences across the habits and age groups (*p*-value < 0.001).

Weekend eating habits across all participants demonstrated significant variation with age (*p*-value < 0.001). The 18–30 age group showed the highest tendency to eat outside, while the 60+ group leaned toward more consistent habits and preparing meals at home (Figure 1).

**FIGURE 2:**
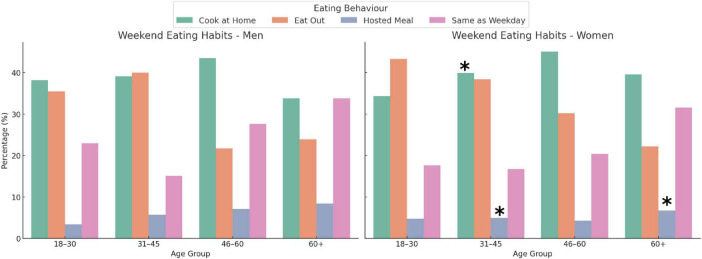
Weekend eating habits by age group and gender. The figure shows the percentage distribution of four distinct responses: I cook elaborate meals at home, I eat out at restaurants or similar, I eat more elaborate meals prepared by others and I eat like on weekdays, among males and females in four age groups (18–30, 31–45, 46–60, 60+). Asterisks above the bars in the female panel indicate statistically significant differences (*p* < 0.05) compared to males in the same age group and response category, as tested with two z-test proportions. Significant within-gender differences were observed for restaurant or similar meals in the 18–30 (*p* = 0.042) and 46–60 (*p* = 0.019) age groups, and for the same or similar weekday meals in the 46–60 group (*p* = 0.034). The within-gender comparison between the age groups showed that, in females, eating out significantly decreased between 18–30 and 46–60 (*p* = 0.015) and between 31–45 and 46–60 (*p* = 0.028), whereas eating the same as weekday meals increased between 18–30 and 46–60 (*p* = 0.044) and between 31–45 and 46–60 (*p* = 0.039). In males, no significant age-related differences were found (all *p* > 0.2).

**FIGURE 3:**
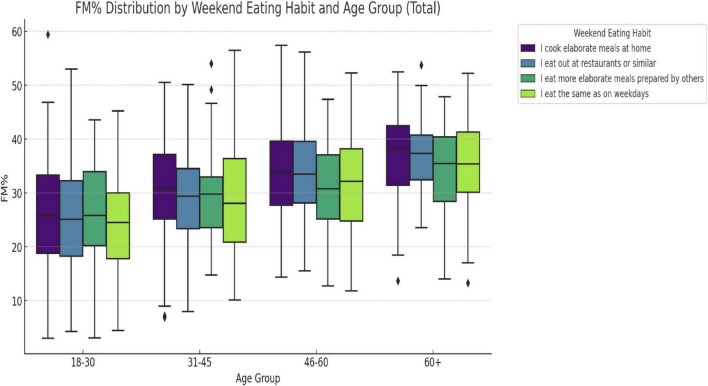
Fat mass (%) distribution by weekend eating habit and age group. The boxplot shows the overall distribution of FM% between weekend eating habits and age groups in the total sample (males and females together). This figure provides a descriptive overview of the differences in FM% according to age and eating habits, without comparing gender. Significant differences were found in the age group 31–45 years (F = 3.12, *p* = 0.025) and in the age group 46–60 years (F = 3.45, *p* = 0.016), with no significant differences in the age groups 18–30 and 60+.

Gender differences in weekend eating habits emerged in specific age groups (Figure 2). Women aged 18–30 were significantly more likely than men to eat out at restaurants or similar (*p* = 0.042). In the 46–60 age group, women reported cooking elaborate meals at home more frequently (*p* = 0.014) and were less likely to maintain the same weekday eating habits (*p* = 0.032). No significant gender differences were observed for the response “I eat more elaborate meals prepared by others” in any age group (all *p* > 0.12). Comparisons within gender between the age groups revealed no significant trends among males (all *p* > 0.2). Figure 3 shows the distribution of fat mass percentage (FM%) across weekend eating habits and age groups in the total sample, highlighting significant differences in the 31–45 and 46–60 age groups. Among women, the frequency of eating out significantly decreased between 18–30 and 46–60 years (*p* = 0.015) and between 31–45 and 46–60 years (*p* = 0.028). In contrast, among females, eating the same as weekday meals increased significantly between 18–30 and 46–60 (*p* = 0.044) and between 31–45 and 46–60 (*p* = 0.039).

**FIGURE 4:**
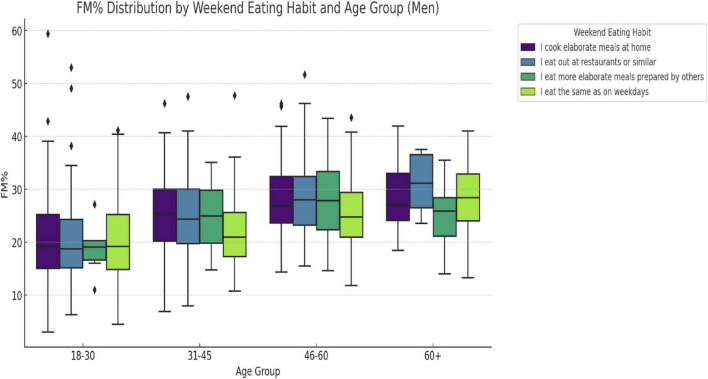
Fat mass (%) distribution by weekend eating habit and age group for males. Fat mass percentage (FM%) distribution by weekend eating behavior and age group in men. “A significant difference in FM% was observed across eating behavior groups in men aged 31–45 years (F = 3.27, *p* = 0.0211).” When comparing age groups within eating behaviors, FM% varied significantly in the “cook at home” (F = 17.22, *p* < 0.0001), “eat out” (F = 23.15, *p* < 0.0001), and “hosted meal” (F = 5.92, *p* = 0.0014) categories.

**FIGURE 5:**
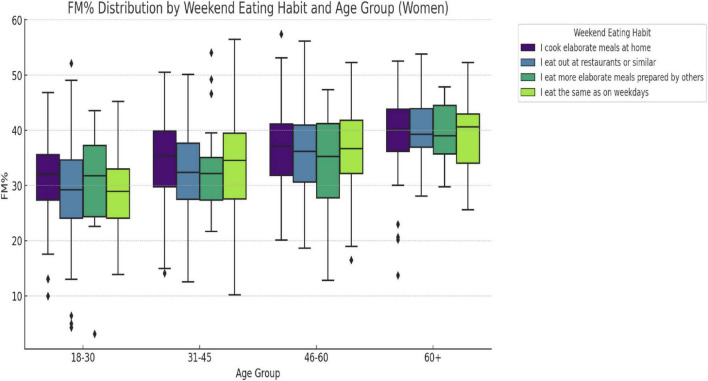
Fat mass (%) distribution by weekend eating habit and age group for females. Fat mass percentage (FM%) distribution by weekend eating behavior and age group in women. “A significant difference in FM% was observed across eating behavior groups in women aged 31–45 years (F = 4.35, *p* = 0.0048).” FM% differed significantly by age only in the “cook at home” group (F = 23.15, *p* < 0.0001).

When stratified by gender and age group (Figures 4, 5), a significant association was observed between weekend eating behavior and FM% in both men (F = 3.27, *p* = 0.0211) and women (F = 4.35, *p* = 0.0048) aged 31–45 years. In these groups, FM% differed significantly between the four reported weekend eating behaviors (“cooking at home,” “eating out,” “eating at home,” and “same as weekdays”). No significant differences in FM% by eating behavior were found among women in the other age groups or among men in the other age groups (all *p* > 0.05). In contrast, when comparing the FM% between the age groups within each eating behavior, significant differences emerged. For the category “cooking at home,” the FM% varied significantly by age in both men (F = 17.22, *p* < 0.0001) and women (F = 23.15, *p* < 0.0001). Among men, FM% also varied significantly between age groups in those who reported “eating out” (F = 23.15, *p* < 0.0001) and “hosting meals” (F = 5.92, *p* = 0.0014) among women who reported having “meals at home” (*p* = 0.0746).

To further explore these differences, a multiple linear regression model was performed with FM% as the dependent variable and gender, age group, and weekend eating behavior as predictors. The analysis confirmed that being female, and being in the 31–45, 46–60, or 60+ age groups, were all significantly associated with higher FM% (*p* < 0.001 for all), while eating out, having hosted meals, or maintaining weekday eating habits were associated with significantly lower FM% compared to cooking at home. Full results are shown in Table 2.

**TABLE 2:** Multiple linear regression analysis of predictors of fat mass percentage (FM%).

Variable	Coefficient	Std. error	*t*	*P*-value	95% CI lower bound	95% CI upper bound
Intercept	21.24	0.41	52.05	< 0.001	20.44	22.04
Gender: female (ref: male)	9.14	0.32	28.74	< 0.001	8.52	9.77
Age group: 31–45 (ref: 18–30)	4.06	0.39	10.29	< 0.001	3.28	4.83
Age group: 46–60 (ref: 18–30)	7.13	0.44	16.29	< 0.001	6.27	7.99
Age group: 60+ (ref: 18–30)	9.46	1.02	15.30	< 0.001	8.24	10.67
Eating habit: eat out (ref: cook at home)	–1.00	0.36	–2.76	0.006	–1.72	–0.29
Eating habit: hosted meal (ref: cook at home)	–1.47	1.13	–2.02	0.044	–2.90	–0.04
Eating habit: same as weekday (ref: cook at home)	–1.72	0.43	–4.03	< 0.001	–2.56	–0.88

Multiple linear regression model with FM% as dependent variable. The independent variables included gender (reference = male), age group (reference = 18–30 years) and weekend eating behavior (reference = cooking at home). Being female and belonging to an older age group were significantly associated with a higher FM%. In contrast, eating out, eating meals at home or maintaining the same eating habits on weekdays were significantly associated with a lower FM% than cooking at home. The coefficients are presented with 95% confidence intervals and *p*-values. The effect of sex reflects both physiological differences in body composition and behavioral differences in weekend eating patterns.

## 4 Discussion

Our results reveal statistically significant gender and age differences in weekend eating habits, reinforcing previous research indicating that eating behavior goes beyond conventional weekday patterns (16). The marked divergence between men and women in meal preparation, meals out and adherence to eating routines suggests that food choices are influenced by a combination of sociocultural, economic and behavioral factors rather than purely individual decisions (17). Women of all age groups showed a significantly greater tendency to prepare meals at home at weekends, while men, especially those aged between 18 and 30, were more likely to eat out. This finding is in line with previous studies showing that, despite changing gender norms, women still take on a greater share of food preparation at home (18). The nutritional implications of this behavior are relevant, as home-cooked meals have been associated with a higher intake of dietary fiber and essential nutrients, potentially contributing to improved long-term health outcomes (16). The greater tendency of women to prepare meals at home may reflect both traditional gender roles and a greater awareness of diet composition and health outcomes. Previous research indicates that women are more engaged in nutrition education and tend to make health-conscious food choices more consistently than men (19).

In contrast, the greater tendency of men, especially younger men, to eat out may be driven by convenience, social factors or limited culinary skills, as suggested by studies linking men’s greater consumption of restaurant and fast food meals to a preference for convenience and lower culinary skills (20). Furthermore, although eating out has been associated with higher total energy intake and higher fat consumption (21), our data showed that women who ate out during the weekend had a lower fat mass percentage than those who cooked at home. This finding may reflect contextual factors, such as stress, time burden, or over-preparation when cooking at home. It also suggests that the social and behavioral dynamics surrounding food preparation play a role in body composition outcomes. Despite changes in social norms, women still have a larger share of culinary responsibilities, although there has been an increase in home food preparation among men in recent years (22). Since the study population was taken from a center specializing in obesity, the sample comprises predominantly overweight or obese individuals. Consequently, the observed weekend eating behavior may not fully reflect that of normal-weight individuals (BMI 20–25), which may differ in terms of psychological, metabolic and lifestyle factors.

Our study highlights how eating habits may evolve with age, particularly in women. These patterns may reflect not only changes related to age but also generational differences in attitudes toward cooking, eating out, and health awareness. Younger women (18–30 years) are more likely to eat out (23), but with advancing age they tend to shift toward home-prepared meals (24). This transition may be influenced by increasing health awareness, family responsibilities or economic factors that reduce dependence on external food sources (24). In contrast, men showed a more stable pattern of eating out across age groups, with older men slightly more likely to maintain similar weekday eating habits (25). This trend is in line with studies that showed that men are less likely to take responsibility for cooking, even in adulthood, and are more dependent on ready-to-eat meals or restaurant food (26). Intervention strategies for younger men should prioritize improving the nutritional profile of restaurant meals and promoting basic cooking skills, as their dependence on eating meals outside the home has been associated with a higher intake of processed foods, saturated fat and sodium (20, 27). Providing accessible training on meal preparation could help reduce these dietary risks and promote healthier choices, as proposed in sustainable dietary strategies (28). These age-related dietary patterns challenge the assumption that eating habits remain stable during adulthood (19). Future longitudinal research should evaluate the evolution of these patterns and their potential impact on metabolic outcomes such as obesity, metabolic syndrome and cardiovascular disease.

Our results indicate that weekend eating habits are associated with differences in body composition, particularly in women. In our study, women who cooked at home during the weekend had a significantly higher FM% than those who ate out, had hosted meals, or maintained weekday eating habits, as confirmed by the multiple regression model. Similarly, among men aged 31–45 and 46–60 years, those who cooked at home had a higher FM% than those who maintained weekday eating patterns (*p* = 0.0028 and *p* = 0.0382, respectively). These findings suggest that home cooking was not necessarily associated with a protective effect on fat mass, as one might expect, and highlight the importance of further investigating the content and context of meals. This trend challenges the assumption that structured or home-based eating automatically results in better body composition. No significant associations were found between weekend eating behavior and fat mass in men overall; however, this should be interpreted with caution, as the survey question did not assess baseline weekday behavior (29–31). It is possible that men in our sample maintained more consistent eating patterns across the week, attenuating contrasts between weekday and weekend behavior. Furthermore, the influence of culturally specific eating habits must be considered, as Italian weekend patterns—such as more home cooking and social meals—may differ from those of other countries, potentially limiting the generalizability of our results to non-Mediterranean populations (12).

This study has several limitations that must be acknowledged. First, relying on self-reported eating behavior may introduce recall bias, as participants may not remember or accurately report their weekend eating habits. Although the sample is large, it comes from a single obesity center in Italy, which may limit the generalizability of the results to larger populations with different dietary patterns and socio-cultural influences. Another limitation is the lack of precise quantitative data on food intake, as we did not assess total caloric intake, macronutrient distribution or meal composition, which could have provided additional information on the relationship between weekend eating behavior and body composition. The design of our study does not allow us to establish causal relationships, as cross-sectional data can only show associations rather than determine whether specific weekend eating habits lead to changes in fat mass percentage over time.

## 5 Conclusion

Weekend eating habits represent a distinct and influential factor on body composition, rather than a simple extension of weekday patterns. The results emphasize that gender- and age-specific approaches are crucial in dietary interventions, particularly for younger men and women who maintain structured meal patterns. Our results suggest that behaviors such as cooking at home at the weekend are not necessarily associated with lower fat mass and may even reflect patterns related to increased adiposity in some subgroups. These findings highlight the need to consider not only the timing and regularity of meals, but also the broader context in which food-related decisions occur. Although our study did not assess food quality or specific dietary content, the frequency and context of cooking at home may be important targets for future interventions. These insights could inform public health strategies aimed at improving long-term metabolic outcomes through better characterization of weekend eating behaviors.

## Data Availability

The raw data supporting the conclusions of this article will be made available by the authors, without undue reservation.
